# Supplementary material for: Synthesis and Biological Evaluation of Cassane Diterpene (5α)-Vuacapane-8(14), 9(11)-Diene and of Some Related Compounds
Source: Molecules. 2022 Sep 4;27(17):5705. doi: 10.3390/molecules27175705 (PMC9458217; doi:10.3390/molecules27175705)
Supplement: Supplementary file 1 [file molecules-27-05705-s001.zip › molecules-1869811-supplementary.pdf]

# Synthesis and Biological Evaluation of Cassane Diterpene (5α)-Vuacapane-8(14), 9(11)-diene and of somes Related Compounds

Zentar Houda[1], Fatin Jannus[2], Marta Medina-O'Donnell[1], José Antonio Lupiáñez[2], José Justicia[1], Ramón Álvarez-Manzaneda[3], Fernando J. Reyes-Zurita[2,*], Enrique Álvarez-Manzaneda[1] and Rachid Chahboun[1,*]

[1] *Departamento de Química Orgánica, Facultad de Ciencias, Instituto de Biotecnología, Universidad de Granada, 18071 Granada*

[2] *Departamento de Bioquímica y Biología Molecular I, Facultad de Ciencias, Universidad de Granada, 18071, Granada*

[3] *Área de Química Orgánica, Departamento de Química y Física, Universidad de Almería, 04120 Almeria, Spain.*

## Table of contents

General information ……………………………………………………………S2

Experimental procedures and characterization data for compounds………………..S3-S8

$^{1}$H and $^{13}$C NMR spectra of compounds ……………..…………………………...S9-S18

1. General information

Unless otherwise stated, reactions were performed in oven-dried glassware under an argon atmosphere using dry solvents. Solvents were dried as follows: benzene over Naebenzophenone, dichloromethane (DCM) over $CaH_2$. Thin-layer chromatography (TLC) was performed using F254 precoated plates (0.25mm) and visualized by UV fluorescence quenching and phosphomolybdic acid solution staining. Flash chromatography was performed on silica gel (230e400 mesh). Chromatography separations were carried out by conventional columnonsilicagel60 (230e400Mesh), usinghexanes- AcOEt (AcOEt-hexane) mixtures of increasing polarity. $^{1}H$ and $^{13}C$ NMR spectrawere recorded at 500 and 400MHz, and at 150,125 and 100MHz, respectively. Chemical shifts (d H) are quoted in parts per million (ppm) referenced to the appropriate residual solvent peak and tetramethylsilane. Data for 1H NMR spectra are reported as follows: chemical shift (d ppm) (multiplicity, coupling constant (Hz), integration), with the abbreviations s, d, t. dd, and m denoting singlet, doublet, triplet, double doublet, and multiplet, respectively. J¼ coupling constant in Hertz (Hz). Data for $^{13}C$ NMR spectra are reported in terms of chemical shift relative to Me4Si (d 0.0) and the signals were assigned utilizing DEPT experiments and on the basis of heteronuclear correlations. Infraredspectra (IR) were recordedas thin films or as solids on a FTIR spectrophotometer with samples between sodium chloride plates and are reported in frequency of absorption (cm_1). Only selected absorbances (nmax) are reported. ([α]D) measurements were carried out in a polarimeter; using a 1dm length cell and $CHCl_3$ as a solvent. The concentration is expressed in mg/mL. HRMS were recorded on a spectrometer, using a Q-TOF analyzer, and ESIþ ionization.

2. Experimental procedures and characterization data for compounds

2.1. *(4bS,8aS)-3-hydroxy-1,4b,8,8-tetramethyl-4b,5,6,7,8,8a,9,10-octahydrophenanthrene-2-carbaldehyde (**20**)*

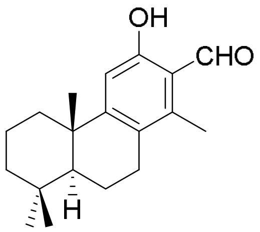


**20**

To a solution of hydroxymetilfenol **19** (113 mg, 0.392 mmol) in dry DCM (10 mL), was added activated $MnO_2$ ( 350 mg, 4.02 mmol) and the reaction mixture was stirred at room temperature for 6 h., at which time TLC showed no starting material. Then, the mixture was filtered through silica gel (10 g) eluted with ethyl acetate. The filtrate was concentrated to give a crude product, which was chromatographed on silica gel (10% AcOEt/hexane) to afford pure salicylic aldehyde **20** (85 mg, 76%) as a colorless oil).

$^1$H NMR ($CDCl_3$, 500 MHz) δ (ppm): 11.84 (s, 1H), 10.40 (s, 1H), 6.82 (s, 1H), 2.82 (ddd, *J* = 16.9, 6.9, 1.5 Hz, 1H), 2.62 (ddd, *J* = 16.9, 11.5, 7.9 Hz, 1H), 2.46 (s, 3H), 2.26 (dd, *J* = 12.7, 1.6 Hz, 1H), 2.00 (ddt, *J* = 13.6, 8.0, 2.0 Hz, 1H), 1.79 (t, *J* = 3.4 Hz, 1H), 1.77 – 1.69 (m, 1H), 1.69 – 1.62 (m, 1H), 1.59 (s, 1H), 1.51 (dtd, *J* = 13.3, 3.3, 1.5 Hz, 1H), 1.44 – 1.33 (m, 1H), 1.33 – 1.24 (m, 1H), 1.22 (s, 3H), 0.99 (s, 3H), 0.96 (s, 3H). $^{13}$C NMR ($CDCl_3$, 125 MHz) δ (ppm): 195.14 (CHO), 161.52 (C), 160.98 (C), 140.19 (C), 125.66 (C), 116.94 (C), 111.27 (CH), 48.96 (CH), 41.37 ($CH_2$), 38.97 (C), 38.88 (CH2), 33.52 (C), 33.17 ($CH_3$), 27.67 ($CH_2$), 24.30 ($CH_3$), 21.71 (CH3), 19.25 ($CH_2$), 18.95 ($CH_2$), 13.35 ($CH_3$). IR (film): 711, 1024, 1217, 1297, 1328, 1368, 1456, 1486, 1571, 1653 cm$^{-1}$.

2.2. *(+)-(4aS,10aS)-7-Isopropyl-6-methoxy-1,1,4a-trimethyl-1,2,3,4,4a,9,10,10a-octahydrophenanthrene (**21**)*

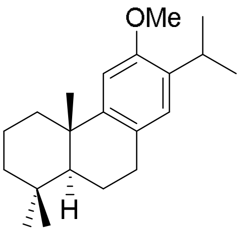


**21**

Potassium carbonate $K_2CO_3$ (41 mg, 0.3 mmol) and iodomethane (100 mg, 0.7 mmol) were added to a solution of ferruginol (**12**) (67 mg, 0.234 mmol) in acetone (3 mL) and the reaction mixture was refluxed for 15 h . Then, the solvent was removed under vacuum and the obtained residue was diluted with ether - $H_2O$ (15: 5 mL) and the phases were shaken and separated. The organic phase was washed with brine (5 mL) and dried over anhydrous $Na_2SO_4$. After filtering and evaporating the solvent, a crude product was obtained and purified by flash chromatography on silica gel (5% EtOAc / hexane) to give compound **21** (56 mg, 85%) as a colorless syrup. $[\alpha]D^{25}$ = +61.9 (c 0.8, $CHCl_3$).

$^1$H NMR ($CDCl_3$, 500 MHz) δ (ppm): 0.95 (3H, s), 0.97 (3H, s), 1.22 (3H, s), 1.23 (6H, d, $J$ = 6.6 Hz), 1.20 - 1.92 (8H, m), 2.28 (1H, m), 2.75 - 2.91 (2H, m), 3.25 (1H, sep, $J$ = 6.6 Hz), 3.82, (3H, s), 6.75 (1H, s), 6.87 (1H, s). $^{13}$C NMR ($CDCl_3$; 125 MHz) δ (ppm): 19.4 ($CH_2$), 19.6 ($CH_2$), 21.8 ($CH_3$), 22.9 ($CH_3$), 23.1 ($CH_3$), 25.0 ($CH_3$), 26.6 (CH), 30.0 ($CH_2$), 33.6 ($CH_3$), 33.7 ($CH_3$), 38.1 (C), 39.1 ($CH_2$), 41.9 (C), 50.7 (C), 55.8, 106.7 (CH), 126.6 (CH), 127.1 (C), 134.3 (C), 148.3 (C), 155.2 (C), IR (film): 1613, 1573, 1499, 1463, 1404, 1374, 1323, 1249, 1207, 1165, 1104, 969, 909, 892, 735 cm-1. HRMS (ESI) (m/z): calcd for $C_{21}H_{32}NaO$ [M + Na]+ 323.2345, found 323.2326.

2.3. *(4bS,8aS)-3-metoxy-4b,8,8-trimethyl-4b,5,6,7,8,8[a],9,10-octahidrophenantrene-2-carbaldehide (**22**):*

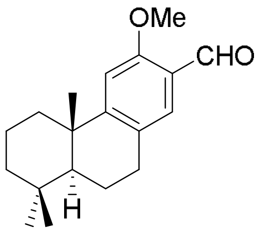

**22**

On a solution of compound **21** (990 g, 3.29 mmol) in anhydrous dichloromethane (20 mL) cooled to -35 ° C and under argon atmosphere, dichloromethyl methyl ether (0.77 ml, 8.5 mmol) was added. To this vigorously stirred mixture, aluminum chloride (1.15 g, 8.62 mmol) was added slowly, in small portions over a period of 5 min. The reaction mixture was then stirred at -35 °C for 15 min. After checking by TLC the complete conversion of compound **22**, the mixture was slowly poured into ice-cold 10% aqueous $NaHCO_3$ and extracted with ethyl acetate (2 x 20 mL). The combined organic layers were washed with water (3 x 10 mL), brine (10 mL), dried over anhydrous $Na_2SO_4$, and filtered. Removal the solvent under vacuum afforded a crude product, which was purified by flash chromatography on silica gel (5% EtOAc / hexane), obtaining aldehyde **22** (0.94 g, 95%) as a colorless syrup. $[\alpha]D^{25}$ = + 11.73 (c = 1.2, $CHCl_3$) $^1$H-RMN ($CDCl_3$, 500 MHz) δ (ppm): 0.94 (s, 3H), 0.94 (s, 3H), 1.22 (s, 3H), 1.30 (dd, *J* = 11.8, 9.4 Hz, 2H). 1.53 – 1.46 (m, 2H), 1.73 – 1.63 (m, 2H), 1.94 – 1.81 (m, 2H), 2.28 (m, 1H), 2.97 – 2.75 (m, 2H), 3.93 (s, 3H), 6.87 (s, 1H), 7.51 (s, 1H), 10.39 (s, 1H). $^{13}$C NMR (125 MHz, chloroform-d) δ (ppm): 18.89. ($CH_2$), 19.20, ($CH_2$), 21.70, ($CH_3$), 24.47 ($CH_3$), 29.21, ($CH_2$), 33.25 (CH), 33.59 (C), 38.75($CH_2$), 38.88 (C), 41.48 (CH2), 49.83 (CH), 55.57 (CH), 107.39 (CH), 122.57 (C), 127.87 (C), 129.01 (CH), 159.03 (C), 160.00 (C), 189.66 (CHO). IR (film): 772, 1038, 1204, 1218, 1263, 1411, 1462, 1608, 1682, 1728, 2867, 2932 $cm^1$. HRMS (ESI) (m/z): calcd for $C_{19}H_{27}O_2$ (M + H+) 287.2011, found 287.2014.

2.4. *(4bS,8aS)-3-hytoxy-4b,8,8-trimethyl-4b,5,6,7,8,8$^a$,9,10-octahydrophenantrene-2-carbaldehide (**23**):*

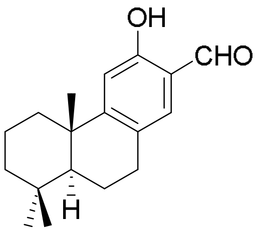

**23**

Over a solution of compound **22** (330 mg, 1.15 mmol) in anhydrous dichloromethane (10 mL) at 0 ° C and under argon atmosphere, aluminum bromide $AlBr_3$ (0.8 g, 3.0 mmol) was slowly added and the reaction mixture was allowed to gradually warm to ambient temperature for 5h. After checking the complete conversion of compound **22** by TLC, Then, the mixture was slowly poured into ice and extracted with ethyl acetate (2 x 20 mL), the combined organic layers were washed with water (3 x 10 mL), brine (10 mL), dried over anhydrous $Na_2SO_4$, and filtered. Removal the solvent under vaccm afforded a crude product, which was purified by flash chromatography on silica gel (10% AcOEt / hexane), yielded phenol **23** (313 mg, 95%) as a white solid. $[\alpha]D^{25}$ = + 2.9 (c= 2.0, $CHCl_3$). $^1$H-RMN (CDCl$_3$, 500 MHz) δ (ppm): 0.94 (s, 3H), 0.94 (s, 3H), 1.22 (s,3H), 1.30 (dd, *J* = 11.8, 9.4 Hz, 2H). 1.53 – 1.46 (m, 2H), 1.73 – 1.63 (m, 2H), 1.94 – 1.81 (m, 2H), 2.29 (m, 1H), 2.97 – 2.75 (m, 2H), 3.93 (s, 3H), 6.87 (s, 1H), 7.51(s, 1H), 10.39 (s, 1H). $^{13}$C-RMN (CDCl$_3$, 125 MHz) δ (ppm): 18.89 ($CH_2$), 19.14 ($CH_2$), 21.72 ($CH_3$), 24.28 ($CH_3$), 29.1 ($CH_2$), 33.3 ($CH_3$), 33.36 (C), 38.56 ($CH_2$), 38.86.3 (C), 41.46 (CH2), 49.64 (CH), 112.99 (CH), 118.8 (C), 127.3 (C), 133.9 (CH), 159.36 (C), 160.84 (C), 195.91 (CHO). IR (film):711, 768, 1023, 1156, 1217, 1297, 1328, 1368, 1456, 1486, 1570, 1651, 2866, 2927 cm$^{-1}$. HRMS (ESI) (m/z): calcd for $C_{18}$H25$O_2$ (M + H+) 273.1855, found 273.1844.

2.5. *(4bS,8aS)-2-(hydroxymethyl)-4b,8,8-trimethyl-4b,5,6,7,8,8a,9,10-octahydrophenanthren-3-ol (**24**)*

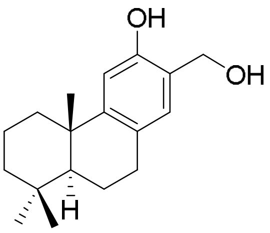

**24**

Sodium borohydride (187 mg, 4.95 mmol) was added to a stirred solution of compound **23** (1.09 g, 4 mmol) in ethanol (15 ml), previously cooled to 0 ° C. The mixture was stirred at room temperature for 1 h. Then, the reaction mixture was cooled at 0 ° C and quenched with sat.aq. $NH_4Cl$ (2 ml). The solvent was removed under vacuum and AcOEt - $H_2O$ (30:10 mL) and the phases were shaken and separated. The aqueous phase was extracted again with AcOEt (2 x 20 mL). The combined organic phases were washed with water (2 x 20 mL), brine (20 mL), dried over anhydrous $Na_2SO_4$ and filtered. Removal of the solvent under vacuum afforded a crude product, which was purified by flash chromatography on silica gel (30% AcOEt/hexane) to yield compound **24** as a white solid (0.85 g,78%). $[\alpha]D^{25}$ = + 20 (c = 0.4, $CHCl_3$). $^1$H NMR (400 MHz, Chloroform-d) δ 0.94 (s, 3H). 0.97 (s, 3H), 1.20 (s, 3H), 1.44 – 1.24 (m, 2H), 1.50 (m, 2H), 1.69 (m, 1H), 1.88 (br dd, *J* = 12.0, 7.4 Hz, 1H), 2.07 (s, 1H), 2.31 – 2.17 (m, 2H), 2.72 (m, 1H), 2.78 (dd, *J* = 11.1, 7.3 Hz, 1H), 2.84 (dd, *J* = 6.9, 2.0 Hz, 1H), 2.88 (dd, *J* = 7.1, 2.0 Hz, 1H), 6.82 (s, 1H), 6.74 (s, 1H). $^{13}$C NMR ($CDCl_3$; 125 MHz) δ (ppm): 19.12 ($CH_2$), 19.30 ($CH_2$), 21.66 ($CH_3$), 24.78 ($CH_3$), 29.53 ($CH_2$), 33.32 ($CH_3$), 33.49 (C), 37.84 (C), 38.83 ($CH_2$), 41.67 ($CH_2$), 50.13 (CH), 64.43 ($CH_2$), 112.35 (CH), 122.13 (C), 126.79 (C), 128.22 (CH), 151.81 (C), 153.78 (C). IR (film): 772, 768, 1023, 1156, 1217, 1247, 1297, 1328, 1368, 1456, 1486, 1570, 1651, 2866, 2927 $cm^{-1}$. HRMS (ESI) (m/z): calcd for $C_{18}H_{25}O_2$ (M - H)- 273.1855, found 273.1866

2.6.*(4aS,11bS)-4,4,11b-trimethyl-2,3,4,4a,5,6,8,11b-octahydrophenanthro[3,2-b]furan-9(1H)-one (**25**)*

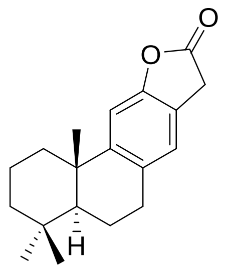

**25**

$Pd(PPh_3)_4$ (72 mg, 0.062 mmol), P(o-tolyl)$_3$ (82 mg, 0.27 mmol) were introduced into a tube and an argon flow was passed inside the tube for 5 min. Then, toluene (1 mL), anhydrous acetic (492 mg, 4.82 mmol), formic acid (235 mg, 5.10 mmol) and, lastly, a solution of **24** (450 mg, 1.64 mmol) in toluene (3 mL) were added. The resulting mixture was sealed and stirred at 100 °C for 16 h, at which time TLC showed the disappearance of compound **24**. The solvent was evaporated under vacuum to afford a crude product, which was purified by flash chromatography on silica gel (20% AcOEt/hexane) to give lactone **25** as a white solid (368 mg, 79%). $[\alpha]D^{25}$ = +32.1 (c 0.5, $CHCl_3$). $^1$H-RMN ($CDCl_3$, 500 MHz) δ (ppm): 0.96 (s, 3H), 0.98 (s, 3H), 1.21 (s, 3H), 1.23 – 1.33 (m, 3H), 1.45 –1.54 (m, 2H), 1.85 - 1.62 (m, 4H), 1.93 (m, 1H), 2.26 (m, 1H), 3.66 (s, 2H), 6.96 (s, 1H), 7.03 (s, 1H). $^{13}$C-RMN ($CDCl_3$, 125 MHz) δ (ppm): 18.94 ($CH_2$), 19.22 ($CH_2$), 21.62 ($CH_3$), 24.79 ($CH_3$), 30.11 ($CH_2$), 32.90 ($CH_2$), 33.25 (CH3), 33.49 (C), 38.28 (C), 39.01 ($CH_2$), 41.56 ($CH_2$), 50.17 (CH), 106.48 (CH), 120.04 (C), 124.91 (CH), 131.04 (C), 151.40 (C), 153.20 (C), 174.75 (C). IR (film): 772, 877, 896, 944, 1004, 1094, 1198, 1376, 1427, 1485, 1800, 2866, 2928, 2957 $cm^{-1}$. HRMS (ESI) (m/z): calcd for $C_{19}H_{25}O_2$ (M + H+) 285.1855, found 285.1843.

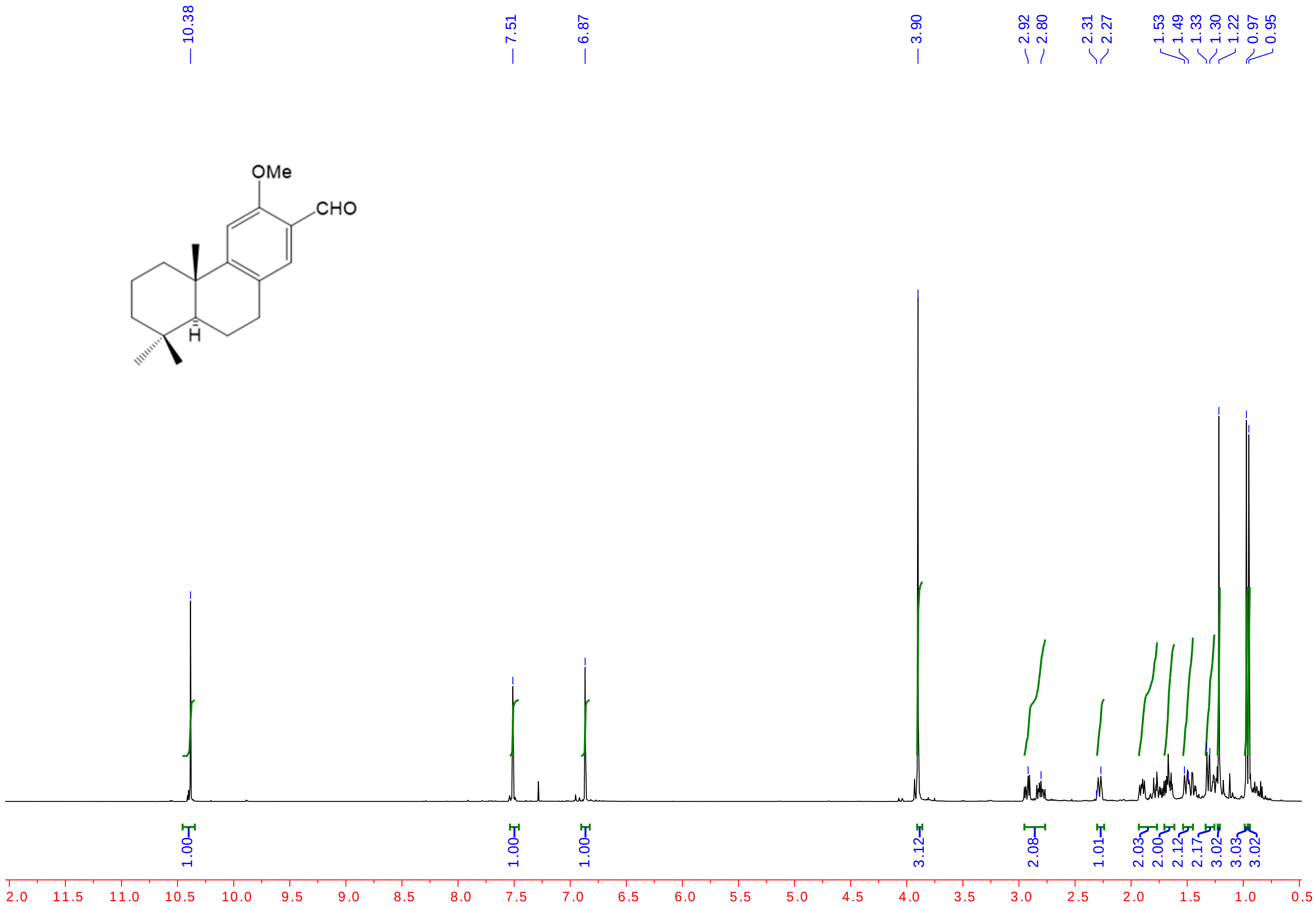
10.38
7.51
6.87
3.90
2.92
2.80
2.31
2.27
1.53
1.49
1.33
1.30
1.22
0.97
0.95
OMe
CHO
H
1.00
1.00
1.00
3.12
2.08
1.01
2.03
2.00
2.12
2.17
3.02
3.03
3.02
2.0 11.5 11.0 10.5 10.0 9.5 9.0 8.5 8.0 7.5 7.0 6.5 6.0 5.5 5.0 4.5 4.0 3.5 3.0 2.5 2.0 1.5 1.0 0.5

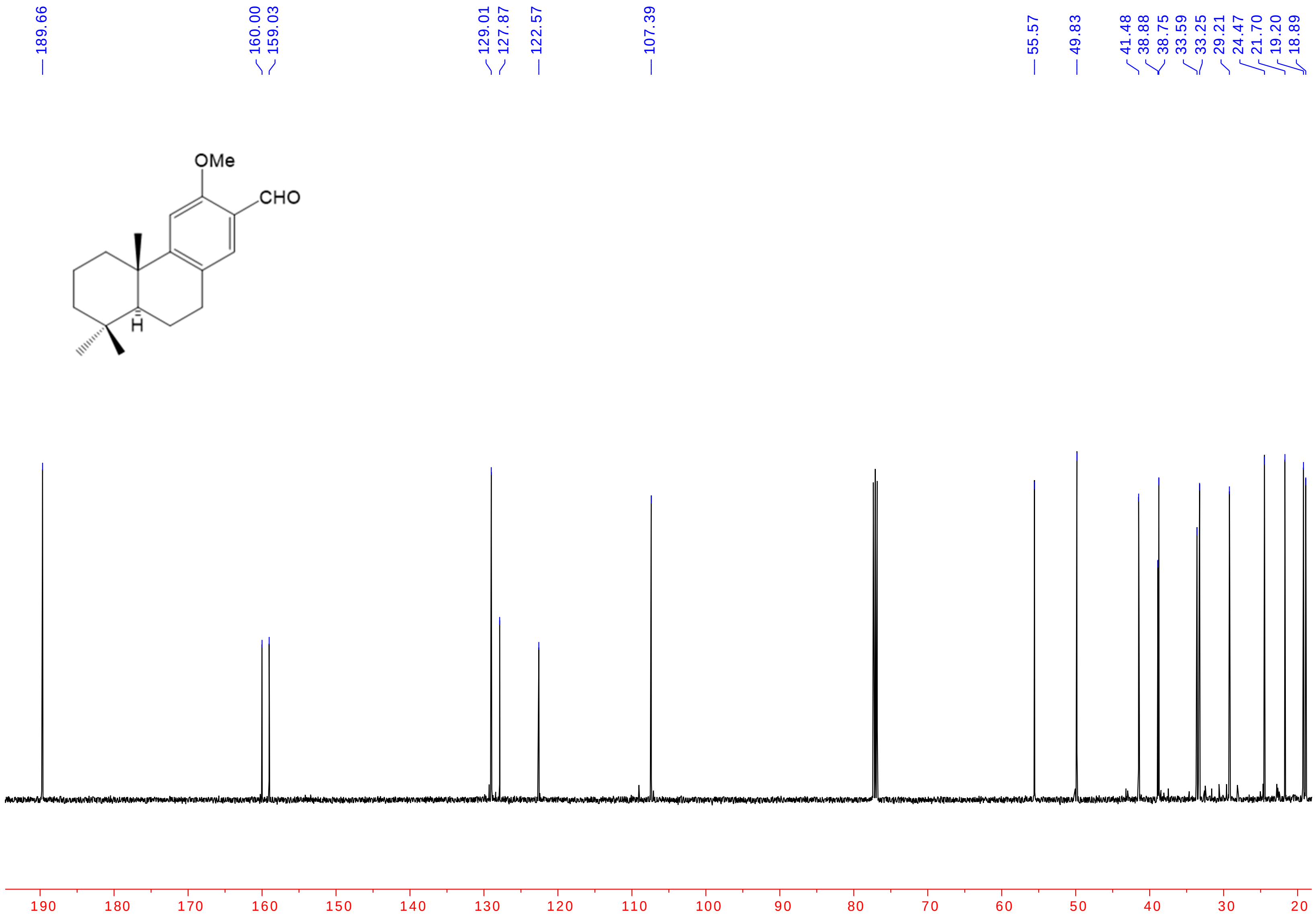
189.66
160.00
159.03
129.01
127.87
122.57
107.39
55.57
49.83
41.48
38.88
38.75
33.59
33.25
29.21
24.47
21.70
19.20
18.89
OMe
CHO
H
190
180
170
160
150
140
130
120
110
100
90
80
70
60
50
40
30
20

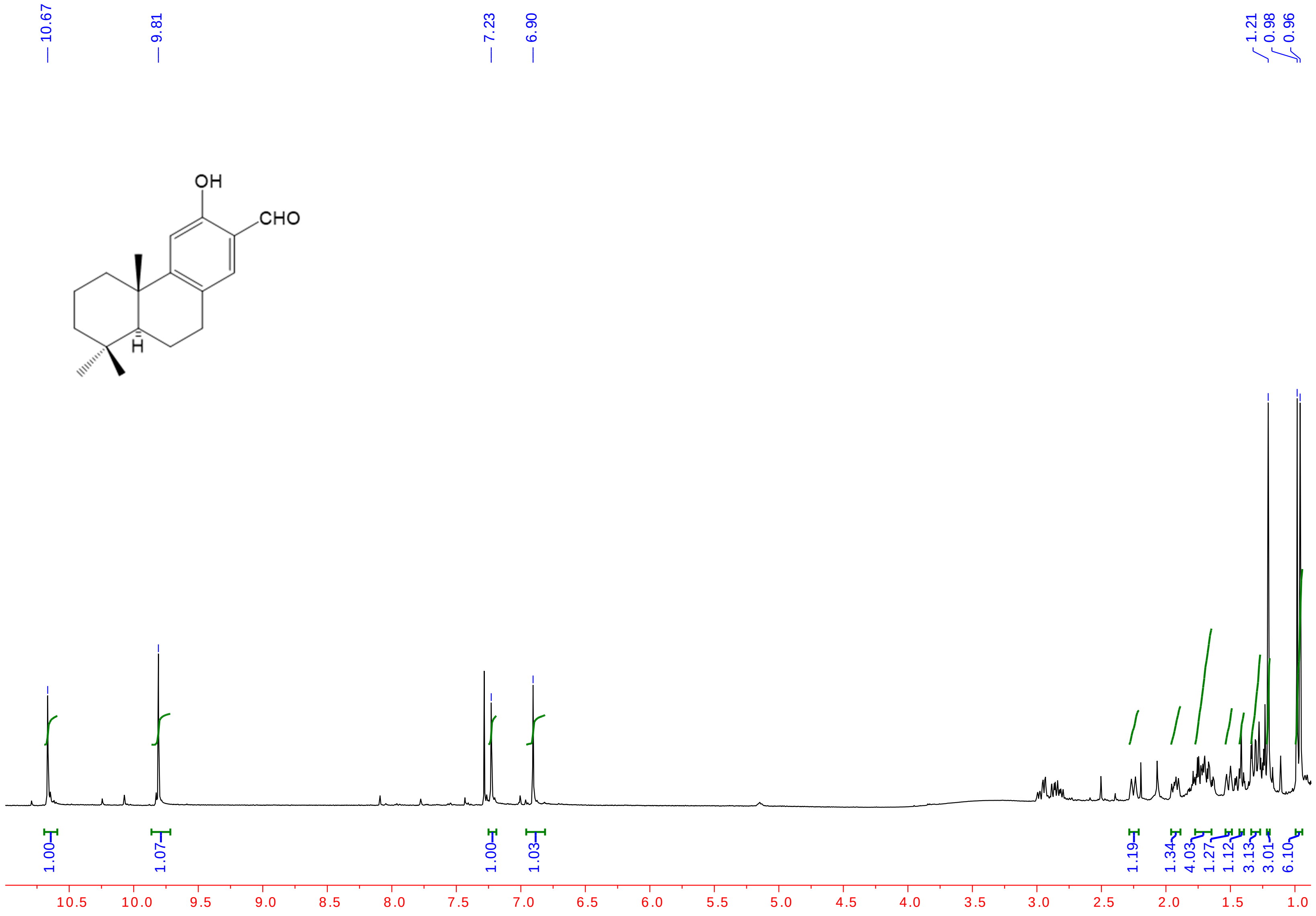
10.67
9.81
7.23
6.90
1.21
0.98
0.96
OH
CHO
H
1.00
1.07
1.00
1.03
1.19
1.34
4.03
1.27
1.12
3.13
3.01
6.10
10.5 10.0 9.5 9.0 8.5 8.0 7.5 7.0 6.5 6.0 5.5 5.0 4.5 4.0 3.5 3.0 2.5 2.0 1.5 1.0

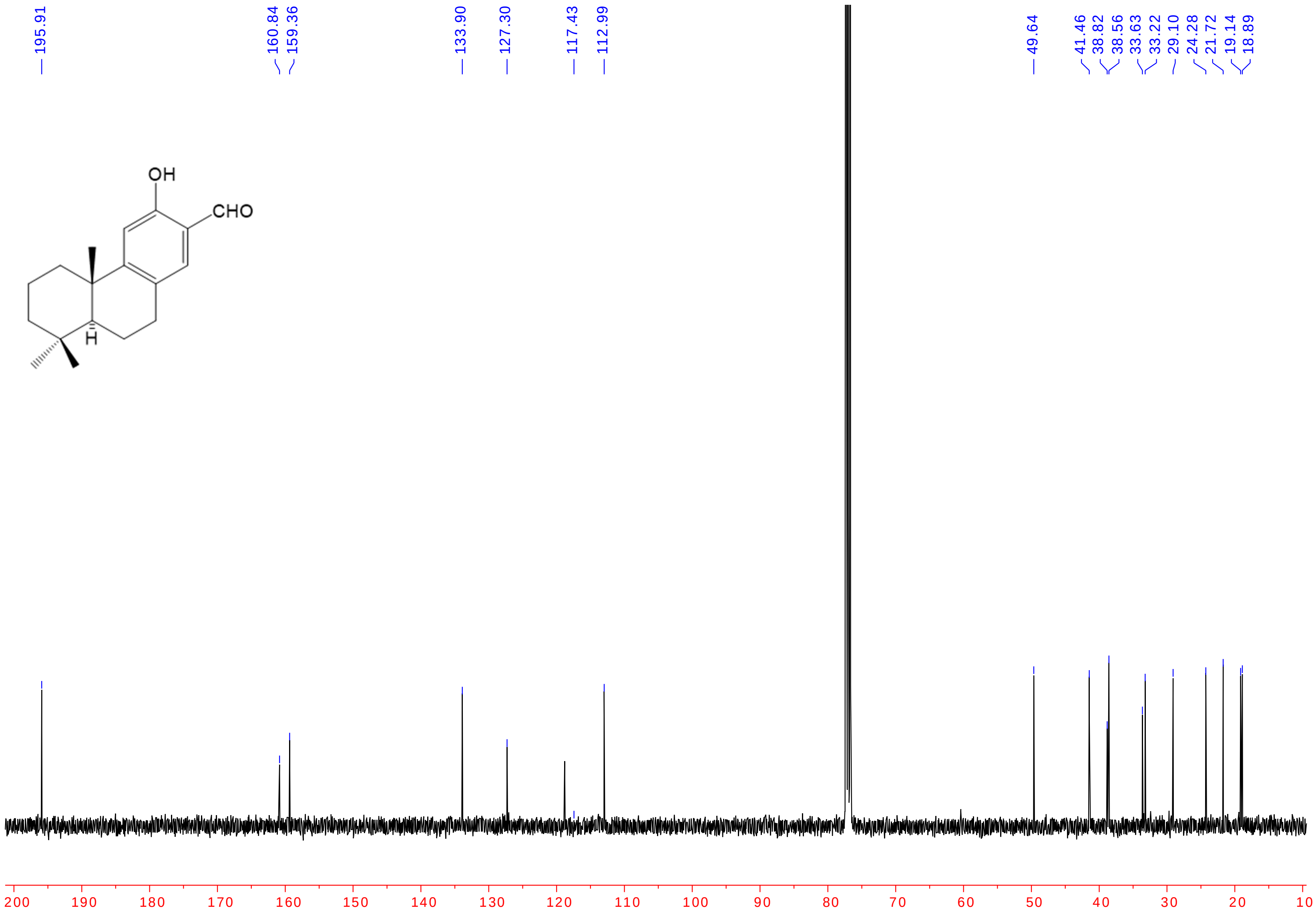
OH
CHO
H
195.91
160.84
159.36
133.90
127.30
117.43
112.99
49.64
41.46
38.82
38.56
33.63
33.22
29.10
24.28
21.72
19.14
18.89
200 190 180 170 160 150 140 130 120 110 100 90 80 70 60 50 40 30 20 10

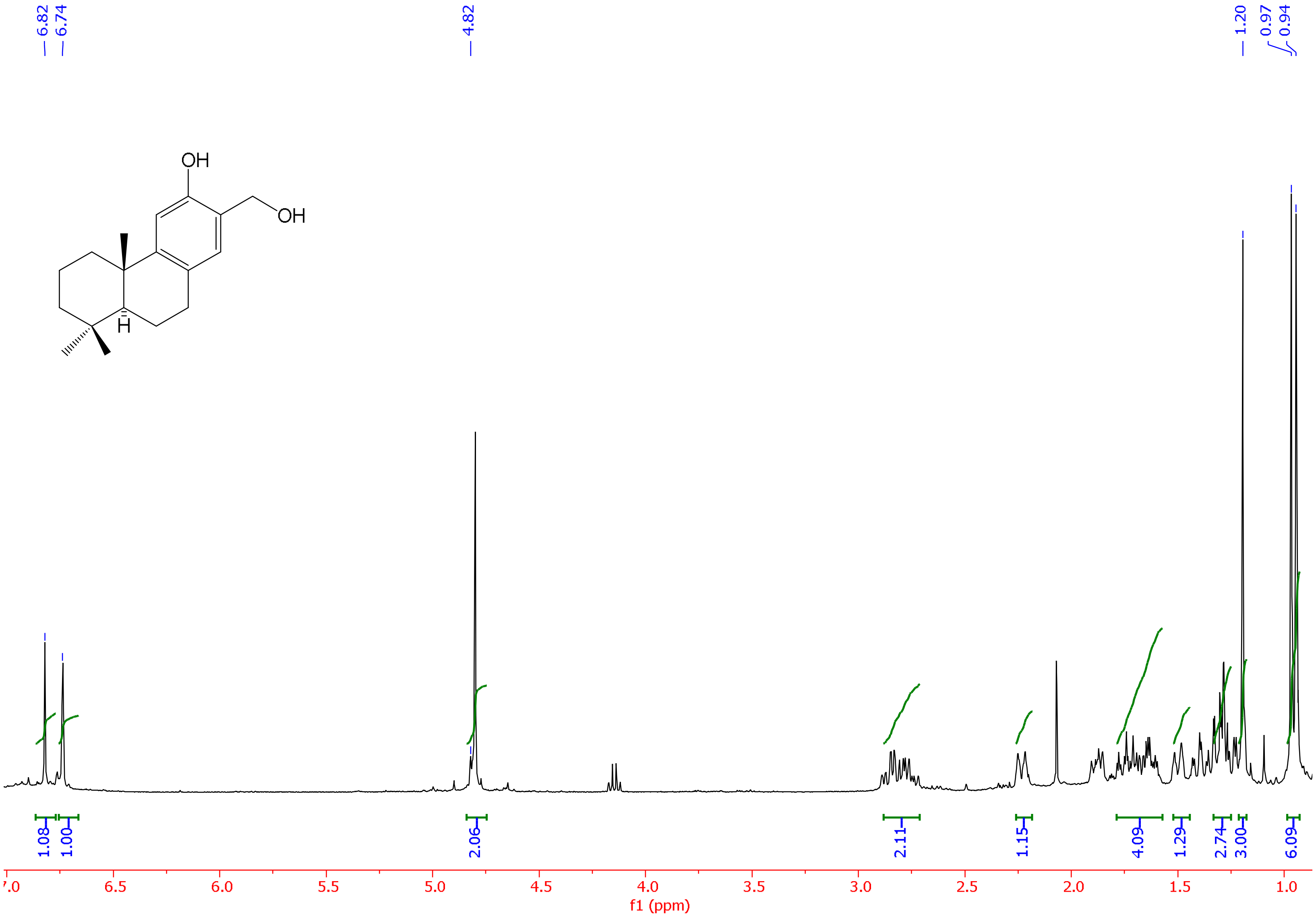

6.82
6.74
4.82
1.20
0.97
0.94
OH
OH
H
1.08
1.00
2.06
2.11
1.15
4.09
1.29
2.74
3.00
6.09
7.0
6.5
6.0
5.5
5.0
4.5
4.0
3.5
3.0
2.5
2.0
1.5
1.0
f1 (ppm)

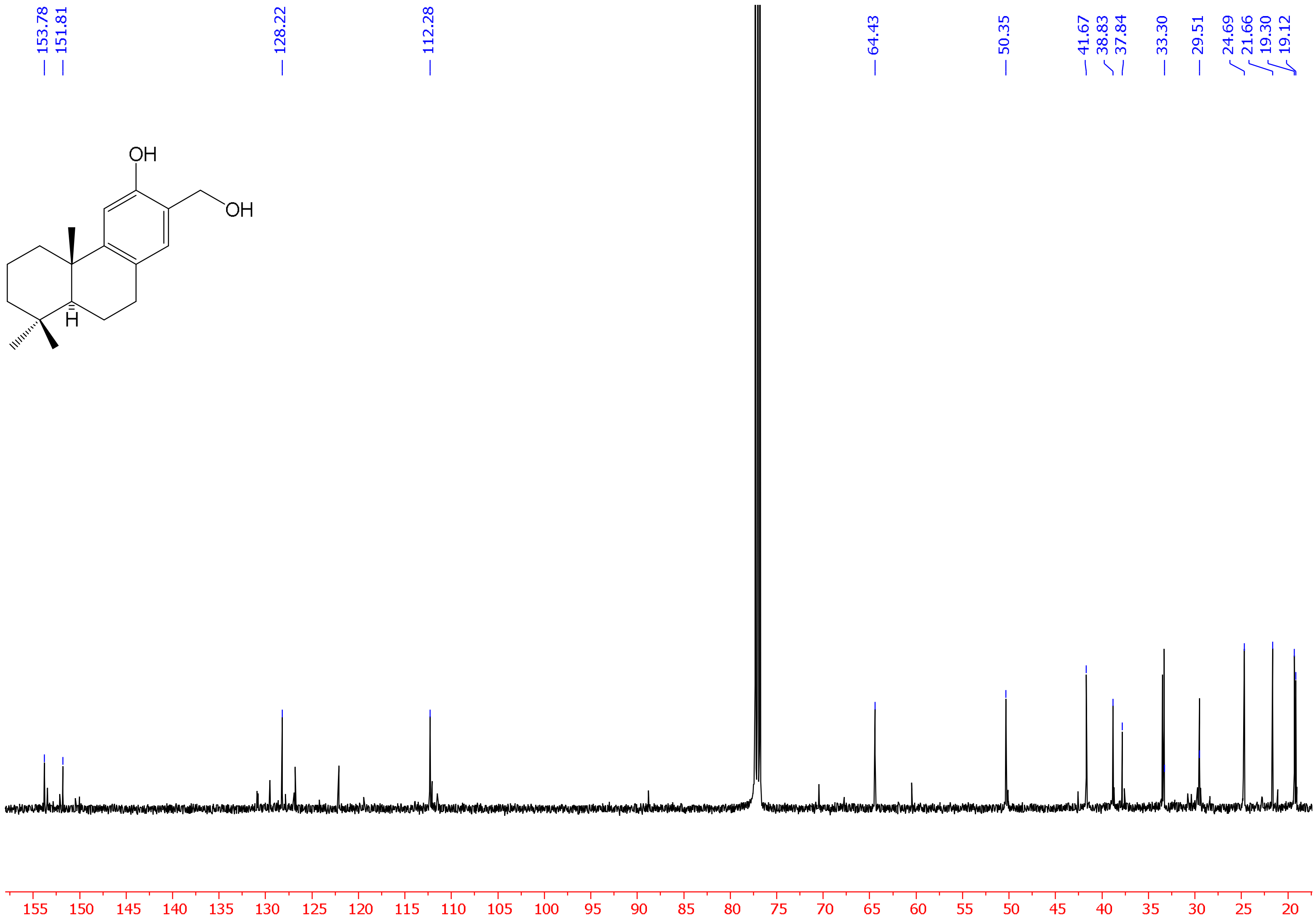
153.78
151.81
128.22
112.28
64.43
50.35
41.67
38.83
37.84
33.30
29.51
24.69
21.66
19.30
19.12
OH
OH
H
155 150 145 140 135 130 125 120 115 110 105 100 95 90 85 80 75 70 65 60 55 50 45 40 35 30 25 20

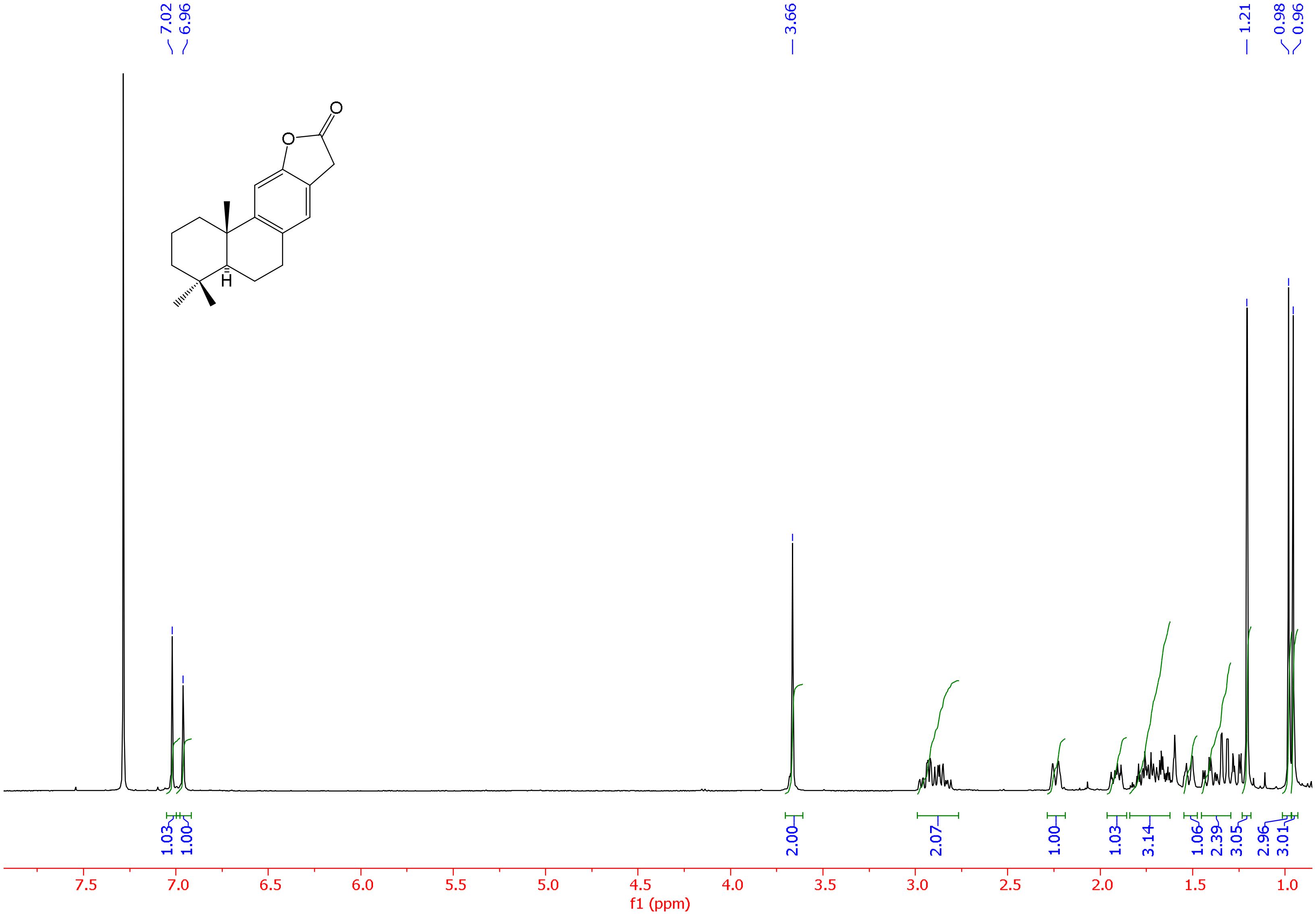
7.02
6.96
3.66
1.21
0.98
0.96
1.03
1.00
2.00
2.07
1.00
1.03
3.14
1.06
2.39
3.05
2.96
3.01
7.5
7.0
6.5
6.0
5.5
5.0
4.5
4.0
3.5
3.0
2.5
2.0
1.5
1.0
f1 (ppm)

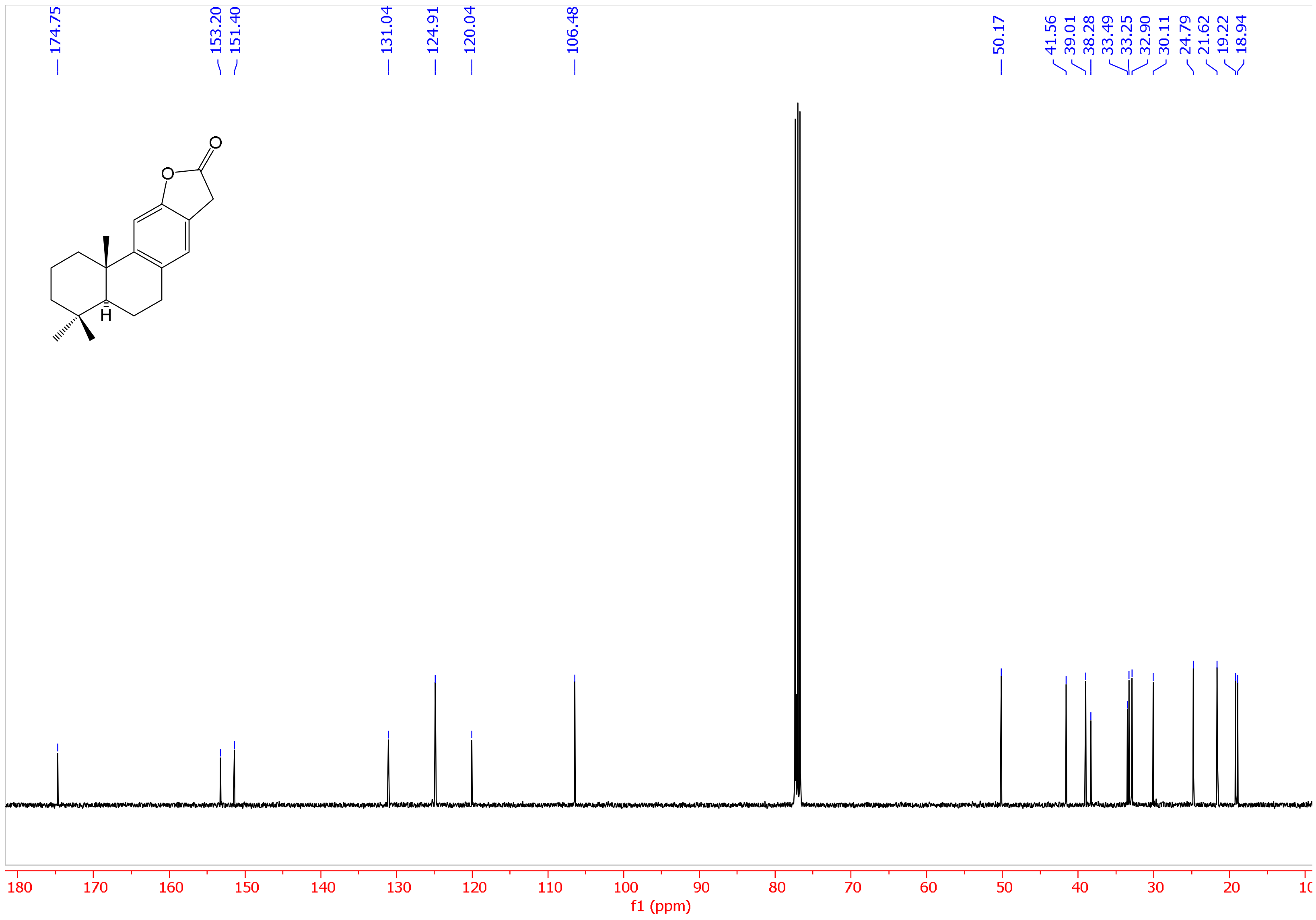
174.75
153.20
151.40
131.04
124.91
120.04
106.48
50.17
41.56
39.01
38.28
33.49
33.25
32.90
30.11
24.79
21.62
19.22
18.94
O
O
O
H
180 170 160 150 140 130 120 110 100 90 80 70 60 50 40 30 20
f1 (ppm)

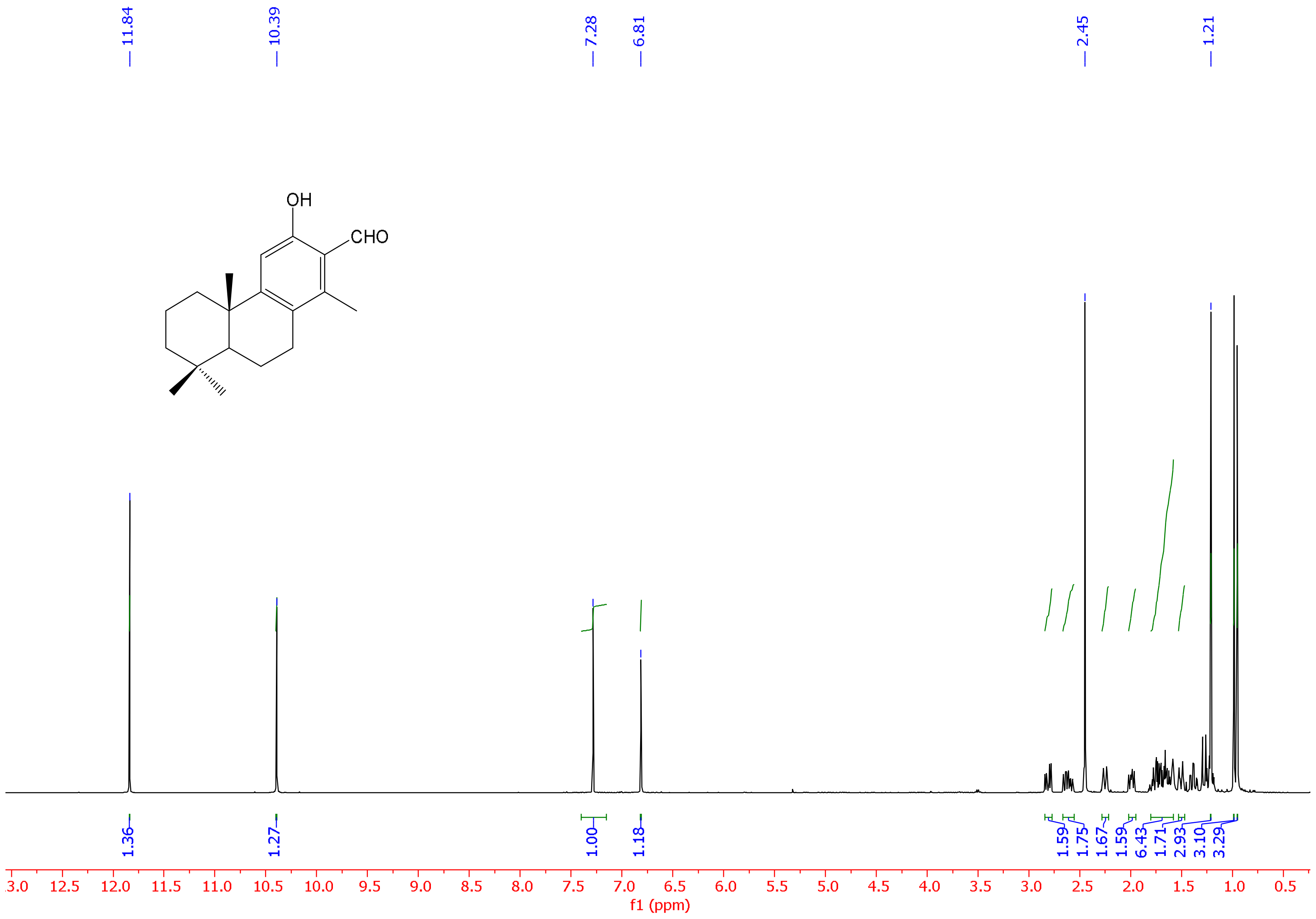
OH
CHO
11.84
10.39
7.28
6.81
2.45
1.21
1.36
1.27
1.00
1.18
1.59
1.75
1.67
1.59
6.43
1.71
2.93
3.10
3.29
3.0 12.5 12.0 11.5 11.0 10.5 10.0 9.5 9.0 8.5 8.0 7.5 7.0 6.5 6.0 5.5 5.0 4.5 4.0 3.5 3.0 2.5 2.0 1.5 1.0 0.5
f1 (ppm)

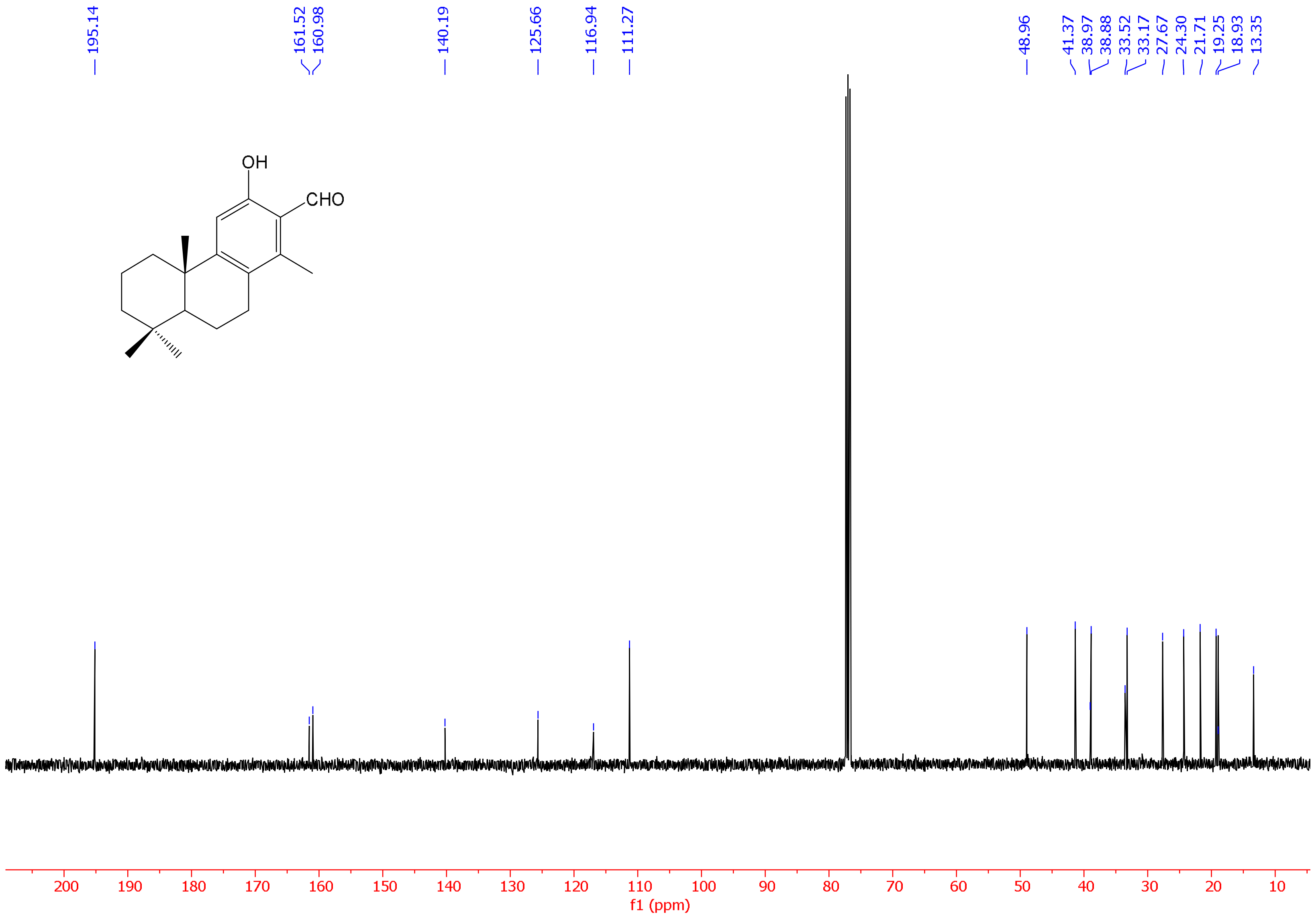
195.14
161.52
160.98
140.19
125.66
116.94
111.27
48.96
41.37
38.97
38.88
33.52
33.17
27.67
24.30
21.71
19.25
18.93
13.35
OH
CHO
f1 (ppm)
